# Causal relationship between the timing of menarche and young adult body mass index with consideration to a trend of consistently decreasing age at menarche

**DOI:** 10.1371/journal.pone.0247757

**Published:** 2021-02-26

**Authors:** Hakyung Kim, Seung-Ah Choe, Soo Ji Lee, Joohon Sung

**Affiliations:** 1 Genome and Health Big Data Laboratory, Department of Public Health, Graduate School of Public Health, Seoul National University, Seoul, Korea; 2 Department of Preventive Medicine, Korea University College of Medicine, Seoul, Korea; 3 Institute of Health & Environment, Seoul National University, Seoul, Korea; University of Mississippi Medical Center, UNITED STATES

## Abstract

Younger age at menarche (AAM) is associated with higher body mass index (BMI) for young women. Considering that continuous trends in decreasing AAM and increasing BMI are found in many countries, we attempted to assess whether the observed negative association between AAM and young adult BMI is causal. We included 4,093 women from the Korean Genome and Epidemiology Study (KoGES) and Healthy twin Study (HTS) with relevant epidemiologic data and genome-wide marker information. To mitigate the remarkable differences in AAM across generations, we converted the AAM to a generation-standardized AAM (gsAAM). To test causality, we applied the Mendelian randomization (MR) approach, using a genetic risk score (GRS) based on 14 AAM-associated single nucleotide polymorphisms (SNPs). We constructed MR models adjusting for education level and validated the results using the inverse-variance weighted (IVW), weighted median (WM), MR-pleiotropy residual sum and outliers test (MR-PRESSO), and MR-Egger regression methods. We found a null association using observed AAM and BMI level (conventional regression; -0.05 [95% CIs -0.10–0.00] per 1-year higher AAM). This null association was replicated when gsAAM was applied instead of AAM. Using the two-stage least squares (2SLS) approach employing a univariate GRS, the association was also negated for both AAM and gsAAM, regardless of model specifications. All the MR diagnostics suggested statistically insignificant associations, but weakly negative trends, without evidence of confounding from pleiotropy. We did not observe a causal association between AAM and young adult BMI whether we considered the birth cohort effect or not. Our study alone does not exclude the possibility of existing a weak negative association, considering the modest power of our study design.

## Introduction

There is good evidence to consider menarche as important for understanding young adulthood obesity [1, 2]. Menarche is a landmark in the female reproductive timespan. It is often preceded by a weight growth spurt during adolescence. A decline in the age at menarche (AAM) has been globally observed for the past 50 years [3–5] coinciding with the obesity epidemic. Girls that experienced early menarche have been observed more prone to overweight/obesity [6]. Previous studies have shown that later spontaneous recovery of obesity established in early life is not common [1, 7]. It is worth noting that, even in the context of dramatic increase in childhood obesity since the 1990s [8], most of obese individuals reported their onset of obesity as age of 17‒18 [1]. Such evidence elicits that young adulthood (18–25 years old) obesity, rather than childhood obesity, substantially influences obesity in later life. It also aligns with the hypothesis that menarche as the marked event of female puberty which occurs at an average age of 12 years, may cause obesity in young adulthood for females.

Mendelian randomization (MR) is an established epidemiological method to infer a causal relationship between risk factors and health outcomes [9], by utilizing a genetic instrumental variable (IV) that is robustly associated with the risk factor. Owing to the random independent assortment of alleles during meiosis, genetic variants with biological effects on risk factors are free from possible confounding factors. Moreover because the transmitted germline genome cannot be affected by health outcomes, it should not be biased by reverse causality [9].

Given that the tendency toward a decrease in AAM is still in progress, identifying the causal relationship between AAM and young adult obesity is crucial for obesity control [10, 11]. This study aimed to assess the causal relationship between AAM and young adult body mass index (BMI). In evaluating the causal association between AAM and young adult obesity, birth cohort effects matter not only for the AAM, but for social determinants such as the length of education. Thus, using these covariates without considerations of birth cohort effects may result in biased estimations. For example, the average AAM decreased from 16.9 years for women born between 1920–1925 and to 13.8 years for those born between 1980–1985 [3]. The proportion of women with higher than upper-secondary education (corresponding to high school in most countries) increased from < 5% for women born in 1926, to 95% for those born in 1970 in South Korea [12]. Given the inverse association between education level and the risk of young adulthood obesity [13, 14], this transitional change in education level may confound the association between AAM and young adult BMI. To address the problem of complex confounding structures, we attempted to introduce a generation-standardized estimation for both AAM and the length of education, using a transgenerational approach. In this study, we estimated the unconfounded causal association of AAM with young adult BMI through the MR approach, using generation-standardized measures.

## Methods

### Study population and variables

The Korean Genome and Epidemiology study (KoGES, http://www.nih.go.kr/NIH/eng/main.jsp) is a community-based prospective cohort study started in 2001, and comprised of 3 sub-cohorts (rural, urban, Ansan-Ansung (KARE)) differing in residential areas of the participants [15]. The Healthy Twin Study (HTS) is a nationwide twin-family cohort study which started in 2005 [16]. Both studies recruited participants of Korean ancestry based on a shared protocol to allow pooled analysis. Detailed study protocols and information have been previously described. From both studies, we only included female participants with epidemiological and genotype information available.

We defined AAM as age in years at the onset of menstruation. Year of birth, age at menarche, and the highest educational attainment, were obtained from self-reported questionnaires. The highest level of educational attainment was categorized into 5 levels: “under elementary school”, “elementary school”, “middle school”, “high school”, and “university/college or higher”. Height measured at enrollment and self-reported body weight at 18–20 years were used to calculate the young adulthood BMI (kg/m^2^). We restricted our analysis to a subset of participants with information on young adulthood BMI. The differences in study variables between the overall population (N = 10,000) and the subset with young adulthood BMI available (N = 4,903) were minimal (S1 Table). The protocol of this study is approved by the Institutional Review Board of Gangnam CHA Hospital (IRB NO: GCI-17-37).

### Defining birth cohorts

We constructed birth cohorts to reflect changes in the distribution of AAM, which has a similar AAM distribution within the year of birth. For example, we started from the birth year of 1927, where the AAM under 14 years was 5.08%, and expanded the birth cohort to include the next recent birth years to as far as the cumulative proportion of AAM under 14 years of the birth cohorts was maintained between 5 and 6%. The end point of the birth cohort was selected when EM at the *K*-birth year showed more than a 2% difference from that of the (*K*+1) birth year. These steps were repeated until the last birth year resulted in four birth cohorts (1927–1945, 1946–1969, 1970–1978, and 1979–2003) (S2 Table).

### Calculating generation-standardized age at menarche (gsAAM) and education

To control for the effect of different birth cohorts, we developed a generation-standardized measure of AAM (gsAAM) and education level. We calculated the z-score of AAM taking individual AAM minus average AAM of the birth year, divided by the standard deviation, where both the averages and standard deviations of AAM were derived from a large population data from the Korea National Health and Nutrition Examination Survey (KNHANES, https://knhanes.cdc.go.kr/knhanes), 2001–2017.

We created an indicator for the relative level of education (“highly educated”) as a proxy for socioeconomic status, to allow us to consider the generation gap in educational attainment (S3 Table). The relative definition of those highly educated within each cohort was varied across birth cohorts: elementary school or higher for 1927–1945, high school or higher for 1946–1969, university/college or higher for both 1970–1978 and 1979–2003 (S1 Fig).

### Selecting genetic markers representing younger AAM

The KoGES and HTS participants were genotyped using Illumina Omni1 (KoGES, rural), Affymatrix 6.0 (KoGES, urban; HTS) and 5.0 (KoGES, KARE) genotyping arrays. The single nucleotide polymorphisms (SNPs) were filtered by following genotype quality control (QC) criteria: (1) genotyping call rate >0.95 (2) minor allele frequency (MAF) >0.01, (3) P value in Hardy–Weinberg equilibrium (HWE) testing >10e^-6^ and individual QC criteria: sample call rate >0.9. Those after QC were imputed using the Korean data from Korean Reference Genome (KRG) which initiated by Center of Genome Science (CGS) of Korea National Instituted of Health (KNIH) in 2012 [17] and East Asian (EAS) data from the 1,000 Genomes Project Phase 3 (NCBI build 37) as the reference. We used IMPUTE 2.0 software to impute variants that were not directly genotyped [18]. Only SNPs with INFO score larger than 0.6 were included.

Among 367 SNPs reported to be associated with AAM from a previous study [19], 297 were available in our genotype data, and 15 SNPs were replicated at a significance level of 0.05 with directional consistency of association. One SNP, rs7132908, located at *FAIM2*, was excluded because it showed a pleiotropic effect with adulthood BMI [20]. Finally, 14 SNPs were selected to build the GRS representing earlier menarche: rs157877 (*RXRG*), rs643428 (*SSBP3*), rs142058842 (*NR4A2*), rs4588499 (*GABRG1*), rs3113862 (*SMARCAD1*), rs1428120 (*GALNT10*), rs13233916 (*TTC26*), rs7115444 (*C11orf67*), rs4945266 (*GAB2*), rs4402316 (*DLG2*), rs7114175 (*C11orf63*), rs3764002 (*WSCD2*), rs10143972 (*UNC79*), rs12915845 (*DET1*) (S4 Table). Childhood BMI, a potential confounder of the effect of AAM on young adulthood BMI, did not show any association at statistical significance (P < 1.0 x 10^−5^) with the selected 14 SNPs according to the GWAS catalogue [21].

### Statistical analysis

#### Validating MR assumptions

We tested whether each SNP included in the GRS and GRS as a whole satisfied the assumptions of the MR method [22]. The underlying assumptions of valid IVs are: 1) the IV has a significant association with the exposure; 2) the IV is not related to any other confounders; and 3) the IV is only related to the outcome through the exposure. F-statistics and R^2^ values were used to ensure that the genetic marker was strongly associated with the risk exposure. Generally, an F-statistic larger than 10 is quoted to reduce the weak instrument bias [23, 24]. The association between risk exposure and GRS quartiles was also examined for convenience of interpretation. To test the second IV assumption of independent association between the genetic IV and young adulthood BMI, the frequencies of potential confounders per risk allele-increase were examined.

#### Conventional regressions and genetic IV-based analyses

For conventional epidemiological analyses, linear regressions were performed to examine the relationship between AAM and young adulthood BMI observationally. We constructed 4 different regression models with varied groupings of birth cohort and education as follows:

Model 1: BMI_young adult_ = AAM_year_ + Year of birth + Educational level + ℇ

Model 2: BMI_young adult_ = AAM_year_ + Birth cohort (defined by AAM distribution) + Educational level (generation-adjusted) + ℇ

Model 3: BMI_young adult_ = gsAAM+ Educational level + ℇ

Model 4: BMI_young adult_ = gsAAM + Educational level (generation-adjusted) + ℇ

Birth cohort variables were treated as dummy variables in the regression models. This analysis was performed using R (The R Foundation, version 3.3.1). We investigated the causal effect of AAM on young adulthood BMI using the two-stage least squares (2SLS) regression, under an additive model assumption through the MR approach [25]. GRS was categorized into quartiles in the first regression of 2SLS for convenience of interpretation. We applied the same four models as in the conventional analyses. The standard error of the 2SLS regressions was obtained by bootstrapping (1000 replications). All MR analyses were performed using STATA version 14 (StataCorp LLC, College Station, Texas).

Implementing the MR analysis without considering pleiotropy effects could lead to bias. To confirm that the IV shows a consistent association with the outcome without the condition of exposure (horizontal pleiotropy), the adjusted MR method using summary data estimation: weighted median(WM) and MR-Egger method using the R package ‘Mendelian Randomization’ [26] and MR-PRSSO using the R package ‘MR-PRSSO’ were employed [27]. The inverse variance weighted (IVW) method calculates the weighted mean using the inverse variance of each SNP to minimize the variance effect [28]. IVW theoretically provides asymptotically equal results from the 2SLS regression when each SNP is fully uncorrelated. The results from IVW are used as a reference compared to other adjusted MR methods. The WM method is a robust estimator that provides consistent estimates even if up to 50% of IVs are invalid [29]. The MR-Egger method is based on Egger’s regression, which is used for adjusting publication bias in meta-analyses [30]. The difference in the intercept term from the origin gives evidence of the average pleiotropic effect in IV used in MR. The coefficient from the MR-Egger regression was used to explore whether the causal effect remains after adjusting for the pleiotropic effect. Scatter plots of summary data estimation were used to graphically interpret the validity of the slope estimates and the intercept of the MR-Egger regression. Cochran’s Q statistics indicating heterogeneity of SNPs [31] were estimated.

To further investigate the influence of AAM on BMI-related traits, we performed additional analyses for non-alcoholic fatty liver disease (NAFLD) and Homeostatic Model Assessment for Insulin Resistance (HOMA-IR). Using the MR-Base (http://www.mrbase.org), a public database and analytic platform of multi-instrument MR [32], the causal association of AAM with NAFLD and HOMA-IR were investigated. Dataset for exposure AAM was selected from GWAS-catalogue [21, 33] and those for NAFLD and HOMA-IR were from prior genome-wide association studies [34–44]. Linkage disequilibrium (LD) clumping method was applied in the process of selecting SNPs to be included. As in our primary analysis, IVW, WM and MR-Egger method was used in two-sample MR analysis.

## Results

In the three Korean cohort studies (KHANES, KoGES, HTS), the proportion of those who reported AAM < 12 was 5–8% in the 1970s group and was 40% in the 1990s group. Mean AAM decreased from 16.6 in women born in 1929–1945 to 12.4 in those born in 1979–1994 (Table 1). We tested the association of educational attainment with young adulthood BMI and AAM (S5 Table and S3 Fig). Overall, AAM decreased with higher educational attainment except the youngest generations (born in 1979―2003). Young adult BMI was lower with higher education attainment groups. Young adulthood BMI was generally higher in the older generations (P for trend < 0.01) (S4 Fig).

**Table 1:** Characteristics of study participants in the Korean Genome and Epidemiology Study (KoGES) and Healthy Twin Study (HTS), (n = 4,093 women).

Birth Cohort	1927–1945 (N = 690)	1946–1969 (N = 2,879)	1970–1978 (N = 367)	1979–2003 (N = 157)	P for difference**	P for trend***
Variables	Mean/N (SD/%)	Mean/N (SD/%)	Mean/N (SD/%)	Mean/N (SD/%)		
Age at menarche, years	16.52 (1.95)	15.18 (1.86)	13.29 (1.35)	12.43 (1.48)	<0.01	<0.01
Generation-standardized age at menarche (z-score)	0.07 (1.05)	-0.01 (1.03)	-0.21 (0.92)	-0.12 (0.98)	<0.01	<0.01
Young adulthood BMI, kg/m^2^	21.84 (3.25)	20.49 (2.56)	19.45 (3.21)	20.61 (4.03)	<0.01	<0.01
University graduation (%)	28 (4.01%)	443 (15.39%)	188 (51.23%)	85 (54.14%)	<0.01	<0.01
Highly educated (%)*	438 (63.48%)	1665 (57.83%)	188 (51.23%)	85 (54.14%)	<0.01	<0.01

SD, Standard deviation; BMI, Body Mass Index.

*Relatively higher education within the cohort, i.e. in 1929–1945, ≥elementary school graduation, in 1946–1969, ≥high school graduation, and in 1970–1994, ≥university graduation.

**Statistical difference test was done with analysis of variance (ANOVA) test for continuous variables and chi-square test for categorical variables.

***Statistical trend test was done with linear regression for continuous variables and Cochran-Armitage trend test for categorical variables

### Assessment of the MR assumptions

We tested GRS to ensure that a higher GRS represents a younger AAM and thus functions as an IV (Table 2). Both AAM in years and gsAAM changed by -0.08 and -0.04 as GRS increased by one unit (both P <0.01). The F-statistics of GRS on AAM was 57.52, indicating that this can be strong IV for younger AAM. The associations between GRS and potential confounders were close to null (S6 Table).

**Table 2:** The regression of AAM on genetic risk score of AAM, the Korean Genome and Epidemiology Study (KoGES) and Healthy Twin Study (HTS) (n = 10,000).

	Coefficients per 1 unit-increase [95% CI]	P	F-statistics	Coefficients per 1 quartile-increase [95% CI]	P	F-statistics
Age at menarche, years	-0.08 [–0.10, -0.06]	<0.01	57.52	-0.11 [–0.14, -0.07]	<0.01	34.48
Generation-standardized age at menarche, z- score	-0.04 [-0.05, -0.03]	<0.01	54.13	-0.05 [-0.07, -0.03]	<0.01	32.50

CI, confidence interval.

### Association of AAM and young adulthood BMI

**Fig 1:**
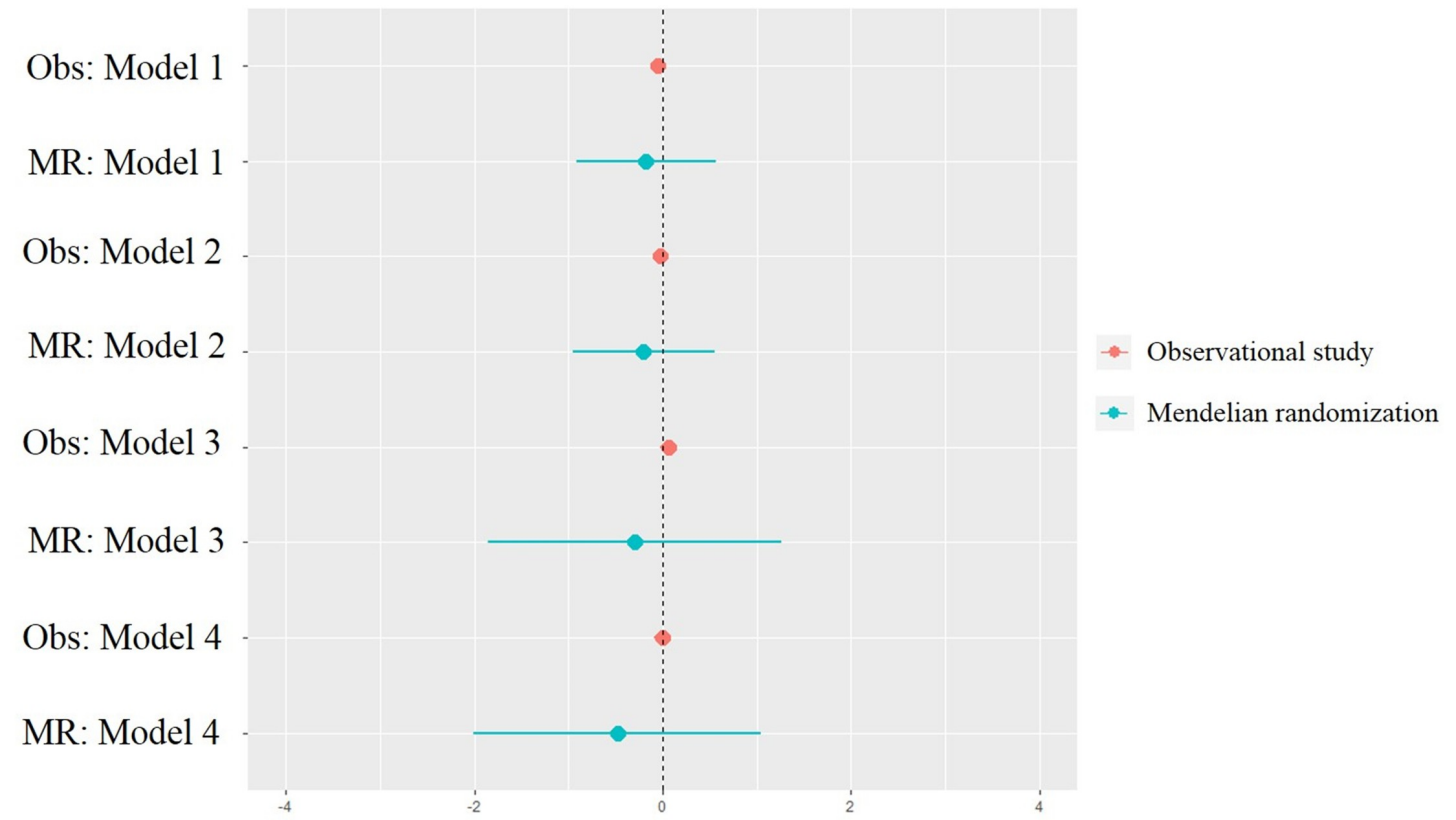
Forest plot of observational analysis and Mendelian randomization analysis. Observational analysis, Obs; Mendelian randomization analysis, MR.

In conventional analyses, higher AAM in year was associated with lower young adulthood BMI (coefficient: -0.05, 95% confidence intervals [CI]: -0.10–0.00; P = 0.04; Table 3). This association was not consistently observed in other models, including those that applied the gsAAM, indicating that the results of conventional analysis could be confounded by how adjustments were selected. In the 2SLS analysis, the associations of both AAM and gsAAM with young adulthood BMI were not demonstrated. However, the results showed consistent trend of negative estimates, in all types of model specifications (Fig 1).

**Table 3:** Association between age at menarche (AAM) and young adulthood body mass index (BMI) using observational and conventional MR analysis, the Korean Genome and Epidemiology Study (KoGES) and Healthy Twin Study (HTS) (n = 4,093 women).

	Observational analysis			Conventional Mendelian Randomization (2SLS)		
	Coefficients [95% CI]	P	R^2^	Coefficients [95% CI]	P	R^2^
Model 1	-0.05 [-0.10, -0.00]	0.04	0.08	-0.18 [-0.91,0.56]	0.64	0.08
Model 2	-0.03 [-0.08, 0.02]	0.28	0.08	-0.20 [-0.95, 0.55]	0.59	0.08
Model 3	0.06 [-0.02, 0.15]	0.15	0.02	-0.30 [-1.85, 1.26]	0.71	0.02
Model 4	-0.003 [-0.09, 0.09]	0.93	0.03	-0.48 [-2.01, 1.04]	0.54	0.03

CI, confidence interval.

Column 1–3 is the result of Observational analysis of AAM and young adulthood BMI and column 4–6 is the result of 2SLS (conventional MR) analysis. Model 1: BMI_young adult_ = AAM_year_ + Year of birth + Education level + ℇ; Model 2: BMI_young adult_ = AAM_year_ + Birth cohort (defined by AAM distribution) + Highly educated + ℇ; Model 3: BMI_young adult_ = gsAAM + Educational level + ℇ; Model 4: BMI_young adult_ = gsAAM + Highly educated + ℇ.

Using the adjusted MR methods (WM, MR-Egger, and MR-PRSSO), we observed larger negative estimates notwithstanding without statistical significances than that from observational studies (Table 4). The intercept estimated from the MR-Egger regression model for AAM (intercept = -0.00, 95% CI: -0.18, 0.18) and gsAAM (intercept = 0.00, 95% CI: -0.18, 0.18) showed no difference from the origin, suggesting that the average pleiotropic effect of SNPs used did not influence the results of the MR analysis. The scatter plot of genetic association with risk factors and outcomes indicated no bias from pleiotropy (S5 Fig). As the Cochran’s Q statistics estimated with 14 SNPs used for GRS indicated heterogeneity between SNPs, we performed the same regression after excluding rs1428120, which showed heterogeneity with other SNPs (S7 Table). The adjusted MR result without rs1428120 was consistent with the result showing heterogeneity.

**Table 4:** Result of adjusted MR method for exploring the association between age at menarche (AAM) and young adulthood body mass index.

	AAM			gsAAM		
MR method	Coefficients [95% CI]	P-value	Cochran’s Q (P value)	Coefficients [95% CI]	P-value	Cochran’s Q (P value)
**Conventional MR**						
**IVW**	-0.71 [-1.71,0.30]	0.17	26.59 (0.01)	-1.27 [-3.04, 0.50]	0.16	26.47 (0.01)
**Adjusted MR**						
**Weighted median (WM)**	-0.48 [-1.55,0.60]	0.38	NA	-0.86 [-0.28,1.04]	0.38	NA
**MR-Egger**	-0.72 [-3.40,1.93]	0.59	26.59 (0.01)	-1.30 [-6.01, 3.46]	0.59	26.47 (0.01)
**(MR-Egger intercept)**	0.00 [-0.18,0.18]	0.99		0.00 [-0.18, 0.18]	0.99	
**MR-PRESSO**	-0.71 [-1.70,0.29]	0.18	NA	-1.27 [-3.04, 0.50]	0.18	NA

IVW, Inverse variance weighted; CI, confidence interval; AAM, age at menarche; gsAAM, generation-standardized age at menarche.

IVW was used as a reference to compare results with adjusted MR methods.

We observed a null association between AAM and NAFLD/HOMA-IR which is consistent with the MR result for adult BMI (S8 Table).

## Discussion

Our study suggests that younger AAM may not be causally associated with BMI in young adulthood. The weak but consistently negative association between the AAM and young adulthood BMI in various MR sensitivity analyses, however, also might suggest a negative association, given the relatively low study power. To reflect the evident secular changes in AAM and educational attainment, we redefined birth cohorts, classifying the participants so that the AAM distributions were similar across birth cohorts. The inverse relationship (earlier menarche associated with higher BMI in young adulthood) reported in observational studies may have been confounded by the secular change of AAM. This notion is supported by the result of 2SLS analysis. Interpretations of the MR analysis requires caution because all the MR models and their adjusted diagnostic models suggest weak but constantly negative associations between AAM and young adulthood BMI. Considering the limited power of the study, we do not conclude that a negative association exist between AAM and young adulthood BMI. Overall, our study suggests a null association, but ours does not exclude the possibility that AAM is one of the determinants of young adulthood obesity, but at least it is not likely that AAM be a major determinant even if so. While the relationships between early puberty and childhood and/or post-pubertal obesity have been investigated, these have not been thoroughly explicated to explain how birth cohort effects may influence the association. To the best of our knowledge, this study is the first to consider generational differences in AAM in exploring the causal association between AAM and young adulthood BMI.

The NAFLD and HOMA-IR are strongly associated with BMI [45, 46]. Also, several observational studies reported that earlier menarche is associated with a higher risk of NAFLD and insulin resistance, independent of young-adult BMI [47, 48]. We tried to explore the causal association of AAM and NAFLD/HOMA-IR in public database. The null association between AAM and NAFLD/HOMA-IR was observed which indicates that AAM does not affect the incident of NAFLD/high HOMA-IR even through obesity.

According to Gill et al. [49], earlier AAM was causally associated with higher adult BMI, even pleiotropic SNPs that were also associated with childhood BMI were excluded. Yet, in another MR study, the inverse association of AAM with BMI at age 18 years was negated when adjusted for childhood BMI [50]. There is another evidence that childhood BMI roles as a confounder of effects of AAM on adulthood BMI [51, 52]. In the 1950s cohort study, the inverse association of AAM with adulthood obesity was not explained by the confounding effect of early childhood BMI [53] and was not replicated in the Australian cohort [54]. The effect of childhood BMI on AAM is somewhat evident but it is still unclear that earlier AAM is one of the cause of young adulthood obesity [55, 56]. The inconsistency between studies may be attributed to the different population distributions in adulthood BMI and AAM.

Given that the trend of AAM in Korea over generations are more dramatically decreased compared to that in western countries and similar patterns are observed worldwide in generational prevalence of childhood obesity [57–59], generational trend of AAM in Korea is not thought to be only caused by increasing childhood obesity. The gap between the trend of AAM and childhood obesity make it necessary to consider the generation effect in the study of causal association between AAM and young adulthood BMI.

The impact of demographic changes over time at a population level has not been explored in previous studies [10, 60]. In the setting of rapid socioeconomic changes, such as in Korea, the historical association between young adulthood obesity and early menarche would not have been evident. We postulated that old Korean women who survived the Korean War had experienced a delayed onset of menarche due to the stressful circumstances, despite most having sufficient body weight to initiate the menstrual cycle [61]. In contrast, in the younger generations, social pressure to maintain a low or normal body weight may have contributed to an overall lower average young adulthood BMI, compared to older women [62, 63]. Individual socioeconomic factors may also confound the association between the timing of menarche. Moreover, SES factors have changed across generations, which further necessitates deciphering the generation effect.

There are a few limitations to this study. First, there may be possibility of recall biases. Those who were obese at the time of the survey might have overestimated their weight in childhood, assuming that they maintained a similar body shape in the past. However, a number of previous studies revealed that self-reported past body weights can provide reliable estimates independent of current weight status [20, 64]. Second, a potential pleiotropic effect may have existed between AAM and young adulthood BMI via residual associations from SNPs not included in the study. As genetic architecture of childhood adiposity and AAM is highly overlapped [19], using subset of AAM SNPs which is only associated with AAM not with childhood adiposity as IV does not assure that the confounding effects of childhood obesity is fully removed. Although we examined most of the established genes and the presence of pleiotropy of IV, there could be a possibility of remaining effect by other genes that were not included in the study. However, we believe this effect would be minimal given the high genetic homogeneity of the Korean population [65]. Third, although our MR results showed consistent negative trend, the association was null and with wide CIs. Both limitations in the size and the strength of the association have resulted in the modest study power, so that further studies are required to confirm the association. Lastly, childhood BMI was not available and could not be included in our analysis. Although we believe the potential confounding role of childhood adiposity was minimized with the use of IV, future studies may benefit from including the information of childhood adiposity.

In conclusion, our findings alone do not exclude the possibility that AAM is one of the determinants of young adulthood obesity, but at least it is not likely that AAM be a major determinant even if so.

## Supporting information

S1 Fig Young adulthood BMI and education attainment across year of birth in the Korean Genome and Epidemiology Study (KoGES) and Healthy Twin Study (HTS), (n = 4,093 women). (DOCX)

S2 Fig Distribution of genetic risk score (AAM) for younger age at menarche (AAM).
(DOCX)

S3 Fig Trend of AAM/young adulthood BMI by educational attainment. (DOCX)

S4 Fig Association between age and young-adulthood BMI. (DOCX)

S5 Fig Scatter plot of adjusted MR estimation. (DOCX)

S1 Table Characteristic comparison of full data (N = 10,000) and data with young adulthood BMI available, Korean Genome and Epidemiology Study (KoGES) and Healthy Twin Study (HTS) (N = 4,093). (DOCX)

S2 Table Definition of birth cohorts based on the distribution of age at menarche (AAM) from the data of Korea National Health and Nutrition Examination Survey (KNHANES), Korean Genome and Epidemiology Study (KoGES) and Healthy Twin Study (HTS) (N = 169,571). (DOCX)

S3 Table Distribution of highest educational attainment by predefined birth cohort from the data of Korean Genome and Epidemiology Study (KoGES) and Healthy Twin Study (HTS) (N = 10,000). (DOCX)

S4 Table SNPs replicated with AAM in the Korean Genome and Epidemiology Study (KoGES) and Healthy Twin Study (HTS), (n = 118,569 women). (DOCX)

S5 Table The association of educational attainment and AAM/young-adulthood BMI. (DOCX)

S6 Table Association of potential confounders with instrumental variable (IV) in the Korean Genome and Epidemiology Study (KoGES) and Healthy Twin Study (HTS), (n = 10,000). (DOCX)

S7 Table Result of two-sample summary MR method for exploring the association between age at menarche (AAM) and young adulthood body mass index without rs1428120. (DOCX)

S8 Table Result of MR-base for exploring the association between age at menarche (AAM) and NAFLD/HOMA-IR. (DOCX)

## References

[1] McTigue KM, Garrett JM, Popkin BM. The natural history of the development of obesity in a cohort of young U.S. adults between 1981 and 1998. Annals of internal medicine. 2002;136(12):857–64. Epub 2002/06/19. 10.7326/0003-4819-136-12-200206180-00006 . 12069559

[2] Braddon FE, Rodgers B, Wadsworth ME, Davies JM. Onset of obesity in a 36 year birth cohort study. British medical journal (Clinical research ed). 1986;293(6542):299–303. Epub 1986/08/02. 10.1136/bmj.293.6542.299 3089493 PMC1340984

[3] Cho GJ, Park HT, Shin JH, Hur JY, Kim YT, Kim SH, et al. Age at menarche in a Korean population: secular trends and influencing factors. Eur J Pediatr. 2010;169(1):89–94. Epub 2009/06/09. 10.1007/s00431-009-0993-1 . 19504269

[4] Nichols HB, Trentham-Dietz A, Hampton JM, Titus-Ernstoff L, Egan KM, Willett WC, et al. From menarche to menopause: trends among US Women born from 1912 to 1969. Am J Epidemiol. 2006;164(10):1003–11. Epub 2006/08/25. 10.1093/aje/kwj282 . 16928728

[5] Bellis MA, Downing J, Ashton JR. Adults at 12? Trends in puberty and their public health consequences. J Epidemiol Community Health. 2006;60(11):910–1. Epub 2006/10/21. 10.1136/jech.2006.049379 17053275 PMC2465479

[6] Bralić I, Tahirović H, Matanić D, Vrdoljak O, Stojanović-Spehar S, Kovacić V, et al. Association of early menarche age and overweight/obesity. J Pediatr Endocrinol Metab. 2012;25(1–2):57–62. Epub 2012/05/11. 10.1515/jpem-2011-0277 . 22570951

[7] Guo SS, Roche AF, Chumlea WC, Gardner JD, Siervogel RM. The predictive value of childhood body mass index values for overweight at age 35 y. The American journal of clinical nutrition. 1994;59(4):810–9. Epub 1994/04/01. 10.1093/ajcn/59.4.810 . 8147324

[8] Ebbeling CB, Pawlak DB, Ludwig DS. Childhood obesity: public-health crisis, common sense cure. Lancet (London, England). 2002;360(9331):473–82. Epub 2002/09/21. 10.1016/S0140-6736(02)09678-2 . 12241736

[9] Smith GD, Ebrahim S. Mendelian Randomization: Genetic Variants as Instruments for Strengthening Causal Inference in Observational Studies. Washington DC: National Academies Press (US); 2008.

[10] Anderson SE, Must A. Interpreting the continued decline in the average age at menarche: results from two nationally representative surveys of U.S. girls studied 10 years apart. The Journal of pediatrics. 2005;147(6):753–60. Epub 2005/12/17. 10.1016/j.jpeds.2005.07.016 . 16356426

[11] Yu EJ, Choe S-A, Yun J-W, Son M. Association of early menarche with adolescent health in the setting of rapidly decreasing age at menarche. Journal of Pediatric and Adolescent Gynecology. 10.1016/j.jpag.2019.12.006 31874313

[12] Yoo SH. Educational differentials in cohort fertility during the fertility transition in South Korea. Demographic Research. 2014;30(53):1463–94.

[13] Watts AW, Mason SM, Loth K, Larson N, Neumark-Sztainer D. Socioeconomic differences in overweight and weight-related behaviors across adolescence and young adulthood: 10-year longitudinal findings from Project EAT. Prev Med. 2016;87:194–9. Epub 03/10. 10.1016/j.ypmed.2016.03.007 . 26970036 PMC4884479

[14] Gonzalez A, Boyle MH, Georgiades K, Duncan L, Atkinson LR, MacMillan HL. Childhood and family influences on body mass index in early adulthood: findings from the Ontario Child Health Study. BMC public health. 2012;12:755. Epub 2012/09/11. 10.1186/1471-2458-12-755 22958463 PMC3490808

[15] Kim Y, Han BG. Cohort Profile: The Korean Genome and Epidemiology Study (KoGES) Consortium. International journal of epidemiology. 2017;46(2):e20. Epub 2016/04/17. 10.1093/ije/dyv316 27085081 PMC5837648

[16] Sung J, Cho SI, Lee K, Ha M, Choi EY, Choi JS, et al. Healthy Twin: a twin-family study of Korea—protocols and current status. Twin research and human genetics: the official journal of the International Society for Twin Studies. 2006;9(6):844–8. Epub 2007/01/27. 10.1375/183242706779462822 . 17254419

[17] Cho YS, Kim H, Kim HM, Jho S, Jun J, Lee YJ, et al. An ethnically relevant consensus Korean reference genome is a step towards personal reference genomes. Nat Commun. 2016;7:13637. Epub 2016/11/25. 10.1038/ncomms13637 27882922 PMC5123046

[18] Howie BN, Donnelly P, Marchini J. A flexible and accurate genotype imputation method for the next generation of genome-wide association studies. PLoS Genet. 2009;5(6):e1000529. Epub 2009/06/23. 10.1371/journal.pgen.1000529 19543373 PMC2689936

[19] Day FR, Thompson DJ, Helgason H, Chasman DI, Finucane H, Sulem P, et al. Genomic analyses identify hundreds of variants associated with age at menarche and support a role for puberty timing in cancer risk. Nature Genetics. 2017;49(6):834–41. 10.1038/ng.3841 28436984 PMC5841952

[20] Casey VA, Dwyer JT, Coleman KA, Krall EA, Gardner J, Valadian I. Accuracy of recall by middle-aged participants in a longitudinal study of their body size and indices of maturation earlier in life. Annals of human biology. 1991;18(2):155–66. Epub 1991/03/01. 10.1080/03014469100001492 . 2024949

[21] Buniello A, MacArthur JAL, Cerezo M, Harris LW, Hayhurst J, Malangone C, et al. The NHGRI-EBI GWAS Catalog of published genome-wide association studies, targeted arrays and summary statistics 2019. Nucleic acids research. 2019;47(D1):D1005–d12. Epub 2018/11/18. 10.1093/nar/gky1120 30445434 PMC6323933

[22] Didelez V, Sheehan N. Mendelian randomization as an instrumental variable approach to causal inference. Stat Methods Med Res. 2007;16(4):309–30. Epub 2007/08/24. 10.1177/0962280206077743 . 17715159

[23] Staiger D, Stock JH. Instrumental Variables Regression with Weak Instruments. Econometrica. 1997;65(3):557–86. 10.2307/2171753

[24] Lawlor DA, Harbord RM, Sterne JA, Timpson N, Davey Smith G. Mendelian randomization: using genes as instruments for making causal inferences in epidemiology. Statistics in medicine. 2008;27(8):1133–63. Epub 2007/09/22. 10.1002/sim.3034 . 17886233

[25] Burgess S, Small DS, Thompson SG. A review of instrumental variable estimators for Mendelian randomization. Statistical Methods in Medical Research. 2015;26(5):2333–55. 10.1177/0962280215597579 26282889 PMC5642006

[26] Yavorska OO, Burgess S. MendelianRandomization: an R package for performing Mendelian randomization analyses using summarized data. International journal of epidemiology. 2017;46(6):1734–9. 10.1093/ije/dyx034 28398548 PMC5510723

[27] Verbanck M, Chen CY, Neale B, Do R. Detection of widespread horizontal pleiotropy in causal relationships inferred from Mendelian randomization between complex traits and diseases. Nat Genet. 2018;50(5):693–8. Epub 2018/04/25. 10.1038/s41588-018-0099-7 29686387 PMC6083837

[28] Burgess S, Bowden J. Integrating summarized data from multiple genetic variants in Mendelian randomization: bias and coverage properties of inverse-variance weighted methods. arXiv preprint arXiv:151204486. 2015.

[29] Bowden J, Davey Smith G, Haycock PC, Burgess S. Consistent Estimation in Mendelian Randomization with Some Invalid Instruments Using a Weighted Median Estimator. Genetic epidemiology. 2016;40(4):304–14. Epub 2016/04/12. 10.1002/gepi.21965 27061298 PMC4849733

[30] Bowden J, Davey Smith G, Burgess S. Mendelian randomization with invalid instruments: effect estimation and bias detection through Egger regression. International journal of epidemiology. 2015;44(2):512–25. 10.1093/ije/dyv080 PMC4469799. 26050253 PMC4469799

[31] Greco MF, Minelli C, Sheehan NA, Thompson JR. Detecting pleiotropy in Mendelian randomisation studies with summary data and a continuous outcome. Statistics in medicine. 2015;34(21):2926–40. Epub 2015/05/08. 10.1002/sim.6522 . 25950993

[32] Hemani G, Zheng J, Elsworth B, Wade KH, Haberland V, Baird D, et al. The MR-Base platform supports systematic causal inference across the human phenome. eLife. 2018;7. Epub 2018/05/31. 10.7554/eLife.34408 29846171 PMC5976434

[33] Horikoshi M, Day FR, Akiyama M, Hirata M, Kamatani Y, Matsuda K, et al. Elucidating the genetic architecture of reproductive ageing in the Japanese population. Nature communications. 2018;9(1):1977–. 10.1038/s41467-018-04398-z . 29773799 PMC5958096

[34] Sun BB, Maranville JC, Peters JE, Stacey D, Staley JR, Blackshaw J, et al. Genomic atlas of the human plasma proteome. Nature. 2018;558(7708):73–9. Epub 2018/06/08. 10.1038/s41586-018-0175-2 29875488 PMC6697541

[35] Astle WJ, Elding H, Jiang T, Allen D, Ruklisa D, Mann AL, et al. The Allelic Landscape of Human Blood Cell Trait Variation and Links to Common Complex Disease. Cell. 2016;167(5):1415–29.e19. Epub 2016/11/20. 10.1016/j.cell.2016.10.042 27863252 PMC5300907

[36] Wu Y, Byrne EM, Zheng Z, Kemper KE, Yengo L, Mallett AJ, et al. Genome-wide association study of medication-use and associated disease in the UK Biobank. Nat Commun. 2019;10(1):1891. Epub 2019/04/25. 10.1038/s41467-019-09572-5 31015401 PMC6478889

[37] Lutz SM, Cho MH, Young K, Hersh CP, Castaldi PJ, McDonald ML, et al. A genome-wide association study identifies risk loci for spirometric measures among smokers of European and African ancestry. BMC Genet. 2015;16:138. Epub 2015/12/05. 10.1186/s12863-015-0299-4 26634245 PMC4668640

[38] Wheeler HE, Gamazon ER, Frisina RD, Perez-Cervantes C, El Charif O, Mapes B, et al. Variants in WFS1 and Other Mendelian Deafness Genes Are Associated with Cisplatin-Associated Ototoxicity. Clin Cancer Res. 2017;23(13):3325–33. Epub 2017/01/01. 10.1158/1078-0432.CCR-16-2809 28039263 PMC5493516

[39] Consortium WTCC. Genome-wide association study of 14,000 cases of seven common diseases and 3,000 shared controls. Nature. 2007;447(7145):661–78. Epub 2007/06/08. 10.1038/nature05911 17554300 PMC2719288

[40] Liu M, Jiang Y, Wedow R, Li Y, Brazel DM, Chen F, et al. Association studies of up to 1.2 million individuals yield new insights into the genetic etiology of tobacco and alcohol use. Nat Genet. 2019;51(2):237–44. Epub 2019/01/16. 10.1038/s41588-018-0307-5 30643251 PMC6358542

[41] Shahin MH, Conrado DJ, Gonzalez D, Gong Y, Lobmeyer MT, Beitelshees AL, et al. Genome-Wide Association Approach Identified Novel Genetic Predictors of Heart Rate Response to β-Blockers. J Am Heart Assoc. 2018;7(5). Epub 2018/02/27. 10.1161/JAHA.117.006463 29478026 PMC5866313

[42] Lauc G, Huffman JE, Pučić M, Zgaga L, Adamczyk B, Mužinić A, et al. Loci associated with N-glycosylation of human immunoglobulin G show pleiotropy with autoimmune diseases and haematological cancers. PLoS Genet. 2013;9(1):e1003225. Epub 2013/02/06. 10.1371/journal.pgen.1003225 23382691 PMC3561084

[43] Mayerle J, den Hoed CM, Schurmann C, Stolk L, Homuth G, Peters MJ, et al. Identification of genetic loci associated with Helicobacter pylori serologic status. Jama. 2013;309(18):1912–20. Epub 2013/05/09. 10.1001/jama.2013.4350 . 23652523

[44] Jensen RA, Sim X, Li X, Cotch MF, Ikram MK, Holliday EG, et al. Genome-wide association study of retinopathy in individuals without diabetes. PLoS One. 2013;8(2):e54232. Epub 2013/02/09. 10.1371/journal.pone.0054232 23393555 PMC3564946

[45] Ahmed ML, Ong KK, Dunger DB. Childhood obesity and the timing of puberty. Trends in endocrinology and metabolism: TEM. 2009;20(5):237–42. Epub 2009/06/23. 10.1016/j.tem.2009.02.004 . 19541497

[46] Chen G, Liu C, Yao J, Jiang Q, Chen N, Huang H, et al. Overweight, obesity, and their associations with insulin resistance and β-cell function among Chinese: a cross-sectional study in China. Metabolism: clinical and experimental. 2010;59(12):1823–32. Epub 2010/07/27. 10.1016/j.metabol.2010.06.009 . 20655552

[47] Mueller NT, Pereira MA, Demerath EW, Dreyfus JG, MacLehose RF, Carr JJ, et al. Earlier menarche is associated with fatty liver and abdominal ectopic fat in midlife, independent of young adult BMI: The CARDIA study. Obesity (Silver Spring). 2015;23(2):468–74. Epub 12/17. 10.1002/oby.20950 . 25521620 PMC4310794

[48] Mueller NT, Duncan BB, Barreto SM, Chor D, Bessel M, Aquino EML, et al. Earlier age at menarche is associated with higher diabetes risk and cardiometabolic disease risk factors in Brazilian adults: Brazilian Longitudinal Study of Adult Health (ELSA-Brasil). Cardiovasc Diabetol. 2014;13:22–. 10.1186/1475-2840-13-22 . 24438044 PMC3899384

[49] Gill D, Brewer CF, Del Greco MF, Sivakumaran P, Bowden J, Sheehan NA, et al. Age at menarche and adult body mass index: a Mendelian randomization study. International journal of obesity (2005). 2018;42(9):1574–81. Epub 2018/03/20. 10.1038/s41366-018-0048-7 . 29549348

[50] Bell JA, Carslake D, Wade KH, Richmond RC, Langdon RJ, Vincent EE, et al. Influence of puberty timing on adiposity and cardiometabolic traits: A Mendelian randomisation study. PLoS medicine. 2018;15(8):e1002641. Epub 2018/08/29. 10.1371/journal.pmed.1002641 30153260 PMC6112630

[51] Kivimäki M, Lawlor DA, Smith GD, Elovainio M, Jokela M, Keltikangas-Järvinen L, et al. Association of age at menarche with cardiovascular risk factors, vascular structure, and function in adulthood: the Cardiovascular Risk in Young Finns study. The American journal of clinical nutrition. 2008;87(6):1876–82. Epub 2008/06/11. 10.1093/ajcn/87.6.1876 . 18541580

[52] Sandhu J, Ben-Shlomo Y, Cole TJ, Holly J, Davey Smith G. The impact of childhood body mass index on timing of puberty, adult stature and obesity: a follow-up study based on adolescent anthropometry recorded at Christ’s Hospital (1936–1964). International journal of obesity (2005). 2006;30(1):14–22. Epub 2005/12/14. 10.1038/sj.ijo.0803156 . 16344844

[53] Pierce MB, Leon DA. Age at menarche and adult BMI in the Aberdeen Children of the 1950s Cohort Study. The American journal of clinical nutrition. 2005;82(4):733–9. 10.1093/ajcn/82.4.733 16210700

[54] Le-Ha C, Beilin LJ, Burrows S, Huang R-C, Hickey M, Mori TA, et al. Age at menarche and childhood body mass index as predictors of cardio-metabolic risk in young adulthood: A prospective cohort study. PLOS ONE. 2018;13(12):e0209355. 10.1371/journal.pone.0209355 30576345 PMC6303033

[55] Chen YC, Fan HY, Yang C, Hsieh RH, Pan WH, Lee YL. Assessing causality between childhood adiposity and early puberty: A bidirectional Mendelian randomization and longitudinal study. Metabolism: clinical and experimental. 2019;100:153961. Epub 2019/08/20. 10.1016/j.metabol.2019.153961 . 31422054

[56] Mumby HS, Elks CE, Li S, Sharp SJ, Khaw KT, Luben RN, et al. Mendelian Randomisation Study of Childhood BMI and Early Menarche. J Obes. 2011;2011:180729. Epub 2011/07/21. 10.1155/2011/180729 21773002 PMC3136158

[57] Meng X, Li S, Duan W, Sun Y, Jia C. Secular Trend of Age at Menarche in Chinese Adolescents Born From 1973 to 2004. Pediatrics. 2017;140(2). Epub 2017/07/19. 10.1542/peds.2017-0085 28716824 PMC5527668

[58] Kim JH, Moon JS. Secular Trends in Pediatric Overweight and Obesity in Korea. Journal of obesity & metabolic syndrome. 2020;29(1):12–7. Epub 2020/03/20. 10.7570/jomes20002 32188238 PMC7118001

[59] Skinner AC, Ravanbakht SN, Skelton JA, Perrin EM, Armstrong SC. Prevalence of Obesity and Severe Obesity in US Children, 1999–2016. Pediatrics. 2018;141(3). Epub 2018/02/28. 10.1542/peds.2017-3459 29483202 PMC6109602

[60] Kaplowitz PB. Link between body fat and the timing of puberty. Pediatrics. 2008;121 Suppl 3:S208–17. Epub 2008/02/15. 10.1542/peds.2007-1813F . 18245513

[61] Choe SA, Sung J. Trends of Premature and Early Menopause: a Comparative Study of the US National Health and Nutrition Examination Survey and the Korea National Health and Nutrition Examination Survey. Journal of Korean medical science. 2020;35(14):e97. Epub 2020/04/14. 10.3346/jkms.2020.35.e97 32281314 PMC7152531

[62] Hosseini M, Kelishadi R, Baikpour M, Ataei N, Qorbani M, Yousefifard M, et al. Age-Period-Cohort Analysis of Obesity and Overweight in Iranian Children and Adolescents. International journal of endocrinology and metabolism. 2017;15(4):e13561. Epub 2018/01/19. 10.5812/ijem.13561 29344031 PMC5750447

[63] Kim Y, Choi S, Chun C, Park S, Khang YH, Oh K. Data Resource Profile: The Korea Youth Risk Behavior Web-based Survey (KYRBS). International journal of epidemiology. 2016;45(4):1076–e. Epub 2016/07/07. 10.1093/ije/dyw070 . 27380796

[64] Must A, Willett WC, Dietz WH. Remote recall of childhood height, weight, and body build by elderly subjects. Am J Epidemiol. 1993;138(1):56–64. Epub 1993/07/01. 10.1093/oxfordjournals.aje.a116777 . 8333427

[65] Jin H-J, Tyler-Smith C, Kim W. The peopling of Korea revealed by analyses of mitochondrial DNA and Y-chromosomal markers. PloS one. 2009;4(1):e4210–e. Epub 01/16. 10.1371/journal.pone.0004210 . 19148289 PMC2615218

