# Supplementary material for: Causal relationship between the timing of menarche and young adult body mass index with consideration to a trend of consistently decreasing age at menarche
Source: PLoS One. 2021 Feb 26;16(2):e0247757. doi: 10.1371/journal.pone.0247757 (PMC7909625; doi:10.1371/journal.pone.0247757)

S1 Fig. Young adulthood BMI and education attainment across year of birth in the Korean Genome and Epidemiology study (KoGES) and Healthy Twin Study (HTS), (n = 4,093 women)


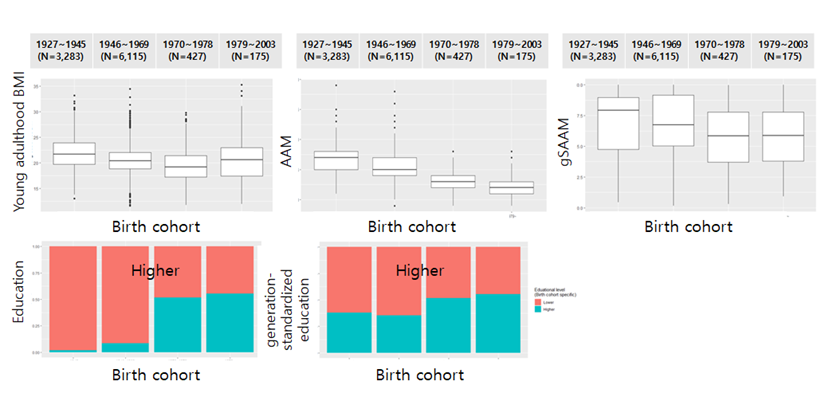

Supplement: S1 Fig — (DOCX) [file pone.0247757.s001.docx]
