# Supplementary figures and images for: Causal relationship between the timing of menarche and young adult body mass index with consideration to a trend of consistently decreasing age at menarche
Source: PLoS One. 2021 Feb 26;16(2):e0247757. doi: 10.1371/journal.pone.0247757 (PMC7909625; doi:10.1371/journal.pone.0247757)

S2 Fig. Distribution of genetic risk score (AAM) for younger age at menarche (AAM)


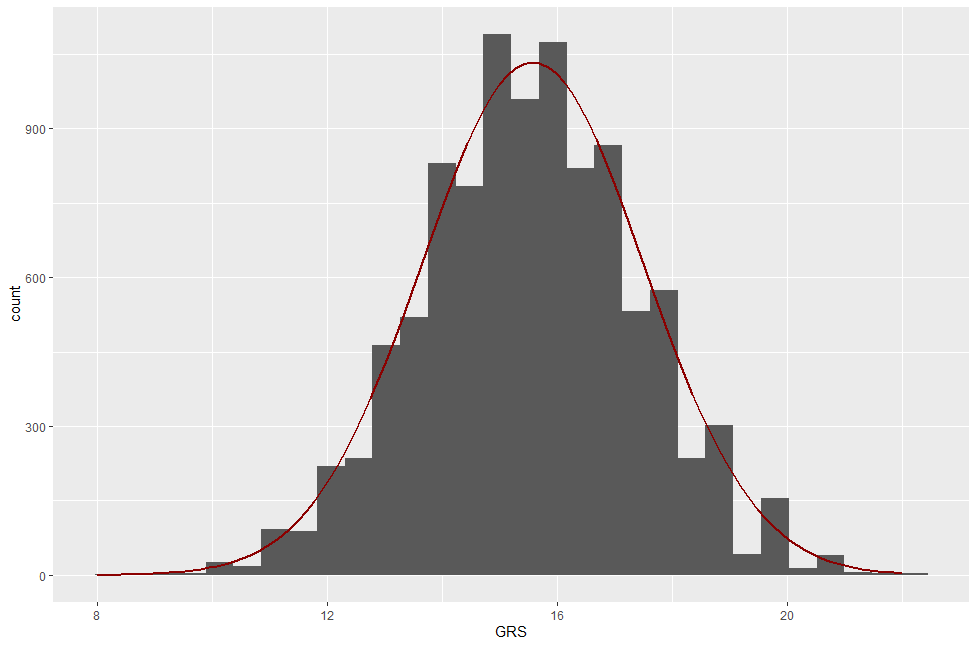

Supplement: S2 Fig — Distribution of genetic risk score (AAM) for younger age at menarche (AAM). (DOCX) [file pone.0247757.s002.docx]

S4 Fig. Association between age and young-adulthood BMI


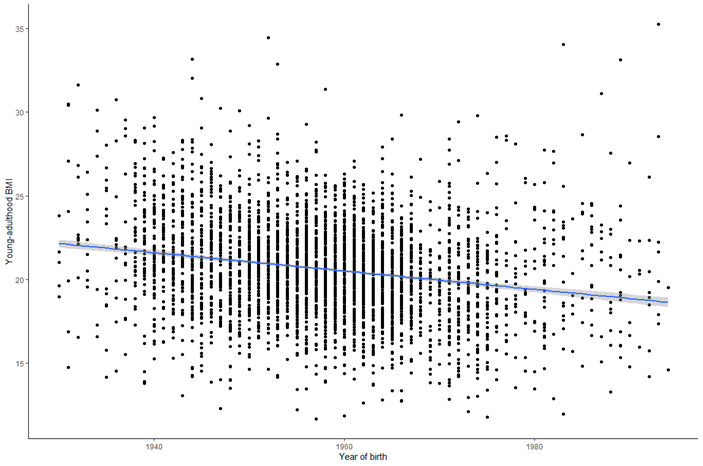

Supplement: S4 Fig — (DOCX) [file pone.0247757.s004.docx]
