# Supplementary material for: Causal relationship between the timing of menarche and young adult body mass index with consideration to a trend of consistently decreasing age at menarche
Source: PLoS One. 2021 Feb 26;16(2):e0247757. doi: 10.1371/journal.pone.0247757 (PMC7909625; doi:10.1371/journal.pone.0247757)
Supplement: S3 Fig — (DOCX) [file pone.0247757.s003.docx]

S3 Fig. Trend of AAM/young adulthood BMI by educational attainment

1. AAM


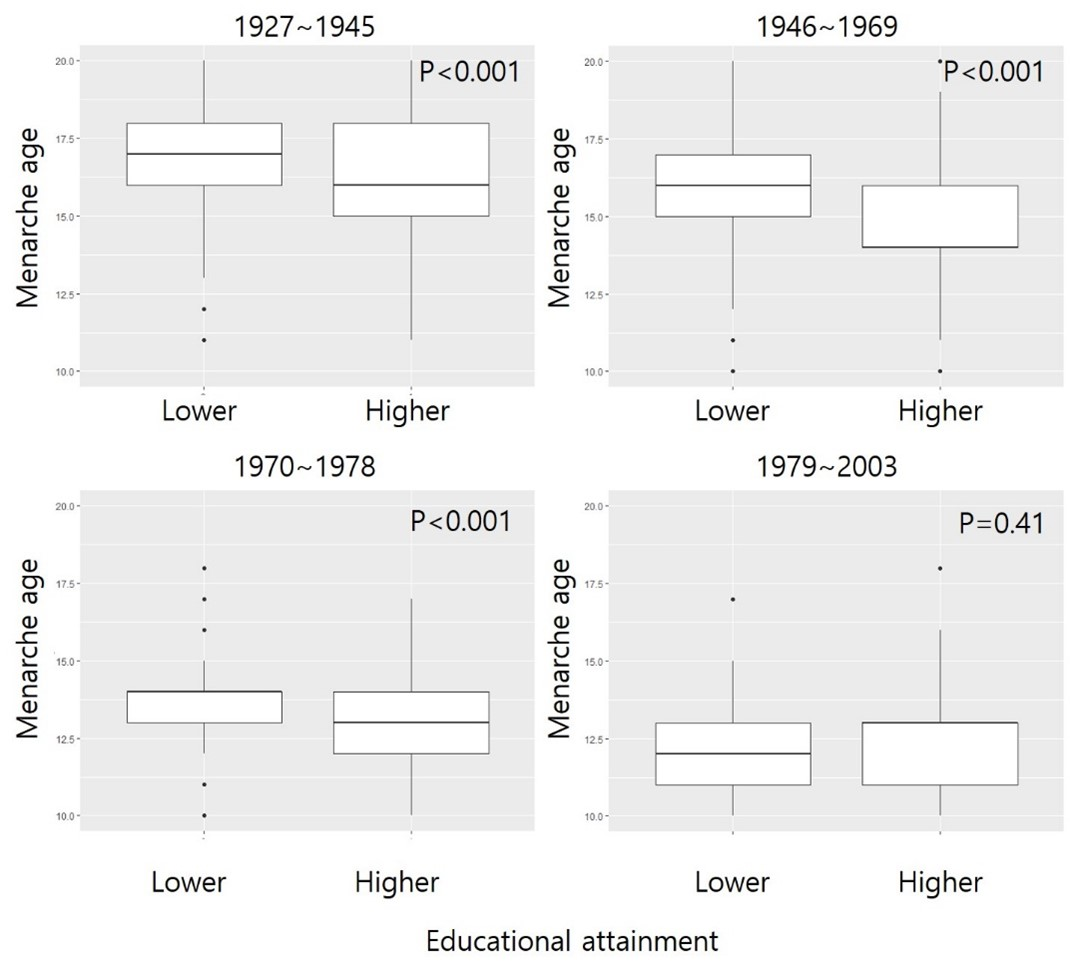


1. Young adulthood BMI


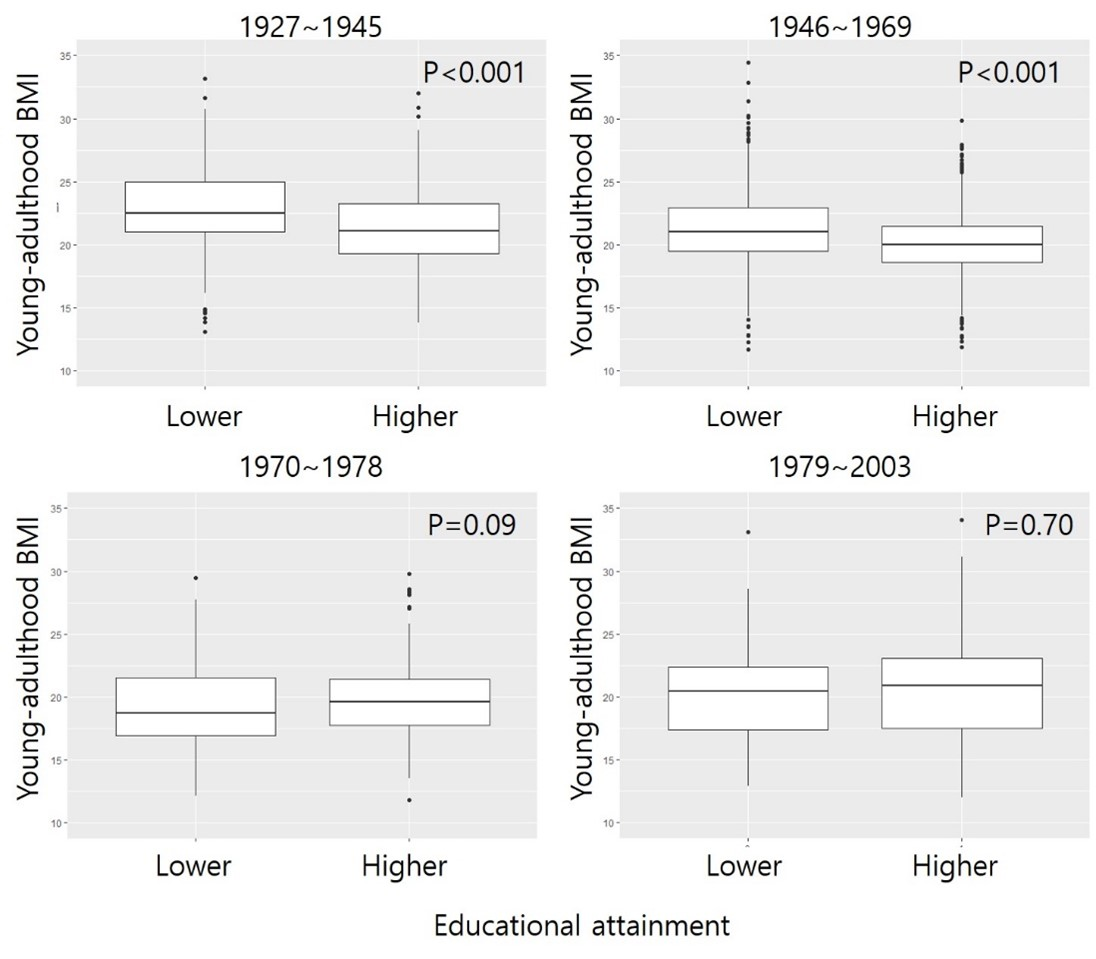


Statistical difference test was done with two-sample t-test.
