# Supplementary material for: Causal relationship between the timing of menarche and young adult body mass index with consideration to a trend of consistently decreasing age at menarche
Source: PLoS One. 2021 Feb 26;16(2):e0247757. doi: 10.1371/journal.pone.0247757 (PMC7909625; doi:10.1371/journal.pone.0247757)
Supplement: S5 Fig — (DOCX) [file pone.0247757.s005.docx]

S5 Fig. Scatter plot of adjusted MR estimation.

Each dot represents each SNP used in the GRS and the line represents the estimated effects by summary data estimation method. Red line is estimation slope of IVW method and Blue line is of MR-egger method.

| 1. Scatter plot of genetic association with young-adult BMI and AAM | 1. Scatter plot of genetic association with young-adult BMI and gsAAM |
| --- | --- |
| 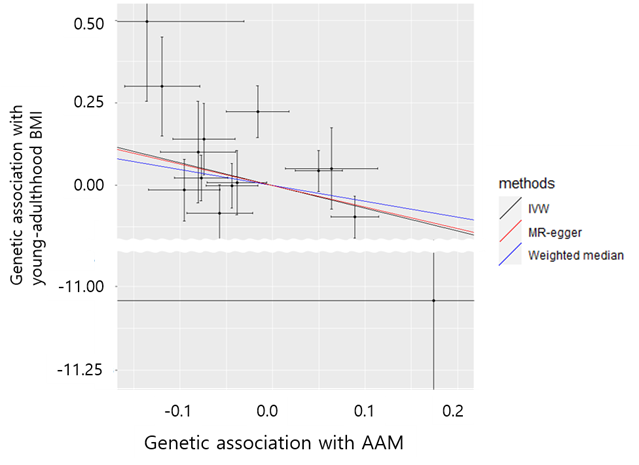 | 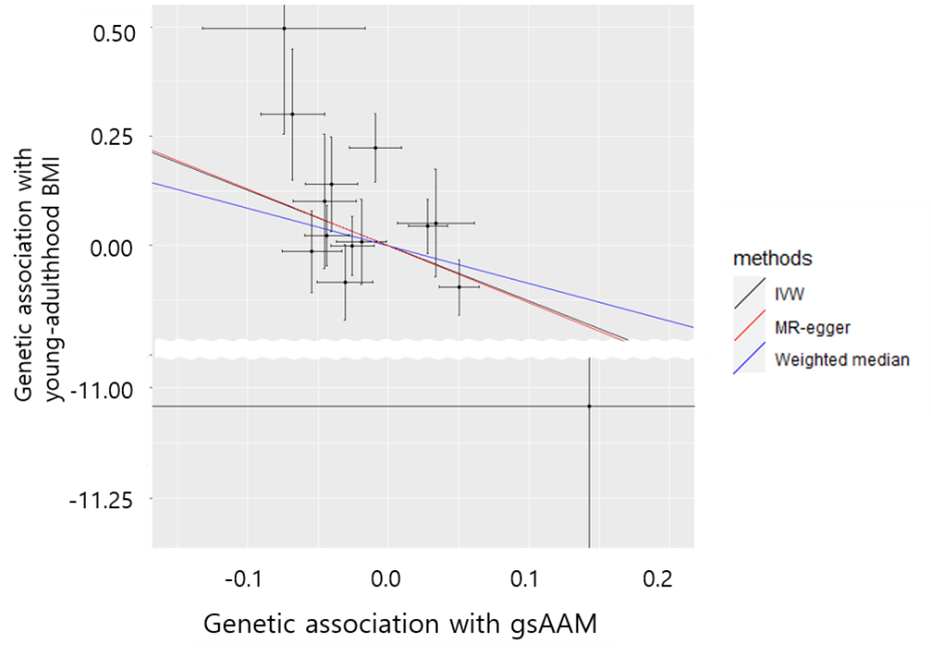 |
