# Supplementary material for: Causal relationship between the timing of menarche and young adult body mass index with consideration to a trend of consistently decreasing age at menarche
Source: PLoS One. 2021 Feb 26;16(2):e0247757. doi: 10.1371/journal.pone.0247757 (PMC7909625; doi:10.1371/journal.pone.0247757)
Supplement: S1 Table — (DOCX) [file pone.0247757.s006.docx]

S1 Table. Characteristic comparison of full data (N=10,000) and data with young adulthood BMI available, Korean Genome and Epidemiology study (KoGES) and Healthy Twin Study (HTS) (N=4,093)

| Birth cohort | 1927-1945 | | | 1946-1969 | | | 1970-1978 | | | 1979-2003 | | |
| --- | --- | --- | --- | --- | --- | --- | --- | --- | --- | --- | --- | --- |
|  | Overall^*^ | Available BMI^**^ |  | Overall^*^ | Available BMI^**^ |  | Overall^*^ | Available BMI^**^ |  | Overall^*^ | Available BMI^**^ |  |
| N | 3,283 | 690 |  | 6,115 | 2879 |  | 427 | 367 |  | 175 | 157 |  |
|  | Mean/N (SD/%) | Mean/N (SD/%) | P | Mean/N (SD/%) | Mean/N (SD/%) | P | Mean/N (SD/%) | Mean/N (SD/%) | P | Mean/N (SD/%) | Mean/N (SD/%) | P |
| AAM | 16.62 (1.89) | 16.52 (1.95) | 0.25 | 15.41 (1.86) | 15.18 (1.86) | <0.01 | 13.33 (1.40) | 13.29 (1.35) | 0.60 | 12.42 (1.48) | 12.43 (1.48) | 0.98 |
| gsAAM | 6.92 (2.28) | 6.78 (2.36) | 0.14 | 6.87 (2.28) | 6.58 (2.37) | <0.01 | 6.26 (2.36) | 6.18 (2.33) | 0.65 | 6.36 (2.29) | 6.36 (2.27) | 0.99 |
| % of university graduation**^***^** | 40 (1.22%) | 28 (4.01%) | <0.01 | 473 (7.74%) | 443 (15.4%) | <0.01 | 221 (51.8%) | 188 (51.2%) | 0.94 | 97 (55.4%) | 85 (54.1%) | 0.90 |
| Highly educated within cohort**^****^** | 1246  (38.0%) | 438 (63.5%) | <0.01 | 2164 (35.4%) | 1665 (57.8%) | <0.01 | 221 (51.8%) | 188 (51.2%) | 0.94 | 97 (55.4%) | 85 (54.1%) | 0.90 |

SD, Standard deviation

^*^Overall (N_total_=10,000)

**Young-adulthood (age of 18-20) BMI available (N_total_=4,093)

* Higher educational attainment of undergrad degree or higher

** Relatively higher education within cohort: 1929-1945 – Over elementary school graduation, 1946-1969- Over high school graduation, 1970-1994- Over university graduation.

Statistical difference test was done with a two sample T-test and a two-sample proportional test.
