# Supplementary material for: Causal relationship between the timing of menarche and young adult body mass index with consideration to a trend of consistently decreasing age at menarche
Source: PLoS One. 2021 Feb 26;16(2):e0247757. doi: 10.1371/journal.pone.0247757 (PMC7909625; doi:10.1371/journal.pone.0247757)
Supplement: S2 Table — (DOCX) [file pone.0247757.s007.docx]

S2 Table. Definition of birth cohorts based on the distribution of age at menarche (AAM) from the data of Korea National Health and Nutrition Examination Survey (KNHANES), Korean Genome and Epidemiology study (KoGES) and Healthy Twin Study (HTS) (N = 169,571)

| Year of birth | Cut-off for earlier menarche (years) | Proportion of early menarche | Total number of women |
| --- | --- | --- | --- |
| 1927-1945 | 14 | 5.79% | 29,171 |
| 1946-1969 | 13 | 5.03% | 120,787 |
| 1970-1978 | 12 | 5.72% | 10,729 |
| 1979-2003 | 11 | 5.64% | 8,884 |
