# Supplementary material for: Causal relationship between the timing of menarche and young adult body mass index with consideration to a trend of consistently decreasing age at menarche
Source: PLoS One. 2021 Feb 26;16(2):e0247757. doi: 10.1371/journal.pone.0247757 (PMC7909625; doi:10.1371/journal.pone.0247757)
Supplement: S3 Table — (DOCX) [file pone.0247757.s008.docx]

S3 Table. Distribution of highest educational attainment by predefined birth cohort from the data of Korean Genome and Epidemiology study (KoGES) and Healthy Twin Study (HTS) (N=10,000)

| Year of birth | 1927-1945 (N=3,283) | 1946-1969 (N=6,115) | 1970-1978 (N=427) | 1979-2003 (N=175) |
| --- | --- | --- | --- | --- |
| Highest education attainment |  |  |  |  |
| Under elementary school | 2037 (62.05%) | 799 (13.07%) | 0 | 0 |
| Elementary | 808 (24.61%) | 1616 (26.43%) | 0 | 0 |
| Middle school | 231 (7.07%) | 1536 (25.12%) | 203 (47.54%) | 3 (1.71%) |
| High school | 143 (4.36%) | 1651 (27.00%) | 150 (35.13%) | 75 (42.86%) |
| University/college or higher | 40 (1.22%) | 473 (7.74%) | 221 (51.76%) | 97 (55.43%) |
| Missing^*^ | 24 (0.73%) | 40 (0.65%) | 0 | 0 |
| Relatively more educated within cohort** | 1245 (37.95%) | 2164 (35.39%) | 221 (51.76%) | 97 (55.43%) |

*Missing value imputed to the most frequent group in the birth cohort in educational level.

**We set generation-adjusted criteria to determine relative level of education within the cohort which comprise high 35-55 percentile: 1929-1945 – Over elementary school graduate, 1946-1969- Over high school graduate, 1970-1994- Over university graduate
