# Supplementary material for: Causal relationship between the timing of menarche and young adult body mass index with consideration to a trend of consistently decreasing age at menarche
Source: PLoS One. 2021 Feb 26;16(2):e0247757. doi: 10.1371/journal.pone.0247757 (PMC7909625; doi:10.1371/journal.pone.0247757)
Supplement: S4 Table — (DOCX) [file pone.0247757.s009.docx]

S4 Table. SNPs replicated with AAM in the Korean Genome and Epidemiology study (KoGES) and Healthy Twin Study (HTS), (n = 118,569 women)

| **Chromosome** | **SNP** | **MAF** | **Replicated coefficient** | **Risk allele** | **Other allele** |
| --- | --- | --- | --- | --- | --- |
| **1** | rs643428 | 0.32 | -0.10 | T | C |
| **1** | rs157877 | 0.14 | -0.15 | A | G |
| **2** | rs142058842 | 0.99 | 0.37 | G | C |
| **4** | rs4588499 | 0.28 | -0.11 | A | G |
| **4** | rs3113862 | 0.24 | -0.10 | A | G |
| **5** | rs1428120 | 0.98 | 0.49 | T | G |
| **7** | rs13233916 | 0.71 | 0.15 | G | C |
| **11** | rs7115444 | 0.55 | 0.12 | T | C |
| **11** | rs4945266 | 0.62 | 0.12 | G | A |
| **11** | rs4402316 | 0.93 | 0.22 | C | G |
| **11** | rs7114175 | 0.70 | 0.11 | T | A |
| **12** | rs3764002 | 0.49 | -0.09 | T | C |
| **14** | rs10143972 | 0.72 | 0.12 | C | T |
| **15** | rs12915845 | 0.12 | -0.15 | T | C |

SNP, Single Nucleotide polymorphism; MAF, Minor Allele Frequency.
