# Supplementary material for: Causal relationship between the timing of menarche and young adult body mass index with consideration to a trend of consistently decreasing age at menarche
Source: PLoS One. 2021 Feb 26;16(2):e0247757. doi: 10.1371/journal.pone.0247757 (PMC7909625; doi:10.1371/journal.pone.0247757)
Supplement: S5 Table — (DOCX) [file pone.0247757.s010.docx]

S5 Table. The association of educational attainment and AAM/young-adulthood BMI

| **Educational attainment** | **AAM** | | **Young adulthood BMI** | |
| --- | --- | --- | --- | --- |
| **Birth cohort** | **Coefficient (SE)** | **P** | **Coefficient (SE)** | **P** |
| 1927~1945 | -0.98 (0.24) | <0.001 | -2.30 | <0.001 |
| 1946~1969 | -0.52 (0.08) | <0.001 | -0.87 | <0.001 |
| 1970~1978 | -0.52 (0.13) | <0.001 | 0.57 (0.33) | 0.09 |
| 1979~2003 | 0.18 (0.22) | 0.41 | 0.25 (0.65) | 0.70 |

SE, Standard Error
