# Supplementary material for: Causal relationship between the timing of menarche and young adult body mass index with consideration to a trend of consistently decreasing age at menarche
Source: PLoS One. 2021 Feb 26;16(2):e0247757. doi: 10.1371/journal.pone.0247757 (PMC7909625; doi:10.1371/journal.pone.0247757)
Supplement: S6 Table — (DOCX) [file pone.0247757.s011.docx]

S6 Table. Association of potential confounders with instrumental variable (IV) in the Korean Genome and Epidemiology study (KoGES) and Healthy Twin Study (HTS), (n = 10,000)

|  | P values for trend (continuous variables) or difference (binary variables) | | | |
| --- | --- | --- | --- | --- |
| SNP | Year of birth | Young adulthood body mass index | Education level (Fixed criteria) | Education level (Relative criteria) |
| rs643428 | 0.33 | 0.94 | 0.07 | 0.17 |
| rs157877 | 0.34 | 0.28 | 0.67 | 0.62 |
| rs142058842 | 0.92 | 0.09 | 0.66 | 0.50 |
| rs4588499 | 0.81 | 0.53 | 0.91 | 0.61 |
| rs3113862 | 0.96 | 0.81 | 0.70 | 1.00 |
| rs1428120 | 0.55 | <0.01 | 0.97 | 0.51 |
| rs13233916 | 0.41 | 0.03 | 0.77 | 0.99 |
| rs7115444 | 0.88 | 0.39 | 0.64 | 0.57 |
| rs4945266 | 0.99 | 0.13 | 0.85 | 0.85 |
| rs4402316 | 0.07 | 0.56 | 0.97 | 0.45 |
| rs7114175 | 0.21 | 0.72 | 0.15 | 0.12 |
| rs3764002 | 0.41 | 0.04 | 0.42 | 0.37 |
| rs10143972 | 0.17 | 0.81 | 0.77 | 0.13 |
| rs12915845 | 0.18 | 0.80 | 0.73 | 0.52 |
| GRS | 0.06 | 0.61 | 0.56 | 0.12 |

SNP, Single Nucleotide polymorphism; GRS, genetic risk score. Statistical analysis was done with linear regression for continuous variables and independent two sample T-test for binary variables.
