# Supplementary material for: Causal relationship between the timing of menarche and young adult body mass index with consideration to a trend of consistently decreasing age at menarche
Source: PLoS One. 2021 Feb 26;16(2):e0247757. doi: 10.1371/journal.pone.0247757 (PMC7909625; doi:10.1371/journal.pone.0247757)
Supplement: S7 Table — (DOCX) [file pone.0247757.s012.docx]

S7 Table. Result of two-sample summary MR method for exploring the association between age at menarche (AAM) and young adulthood body mass index without rs1428120

|  | AAM | | | gsAAM | | |
| --- | --- | --- | --- | --- | --- | --- |
| Method | Coefficients [95% CI] | P-value | Cochran’s Q (P-value) | Coefficients [95% CI] | P-value | Cochran’s Q  (P value) |
| Conventional MR | | | | | | |
| IVW | -0.69  [-1.57, 0.18] | 0.12 | 18.74 (0.09) | -1.23  [-2.78, 0.32] | 0.12 | 18.71 (0.10) |
| Adjusted MR | | | | | | |
| Weighted median | -0.48 [-1.54, 0.89] | 0.38 | - | -0.86 [-2.74, 1.02] | 0.38 | - |
| MR-Egger | -0.65  [-2.98, 1.67] | 0.58 | 18.74 (0.07) | -1.10  [-5.28 , 3.09] | 0.61 | 18.71 (07) |
| (MR-Egger intercept) | -0.00  [-0.16, 0.15] | 0.97 |  | 0.01  [-0.17, 0.16] | 0.94 |  |

IVW, inverse-variance weighted; CI, confidence interval.
