# Supplementary material for: Causal relationship between the timing of menarche and young adult body mass index with consideration to a trend of consistently decreasing age at menarche
Source: PLoS One. 2021 Feb 26;16(2):e0247757. doi: 10.1371/journal.pone.0247757 (PMC7909625; doi:10.1371/journal.pone.0247757)
Supplement: S8 Table — (DOCX) [file pone.0247757.s013.docx]

S8 Table. Result of MR-base for exploring the association between age at menarche (AAM) and NAFLD/HOMA-IR

|  | NAFLD | | | HOMA_IR | | |
| --- | --- | --- | --- | --- | --- | --- |
| Method | Coefficients [95% CI] | P-value | Cochran’s Q (P-value) | Coefficients [95% CI] | P-value | Cochran’s Q  (P value) |
| IVW | -0.17 [-0.66,0.32] | 0.50 | 7.40 (0.49) | 0.001 [-0.02, 0.02] | 0.92 | 4.49 (0.81) |
| Weighted median | -0.22 [-0.86,0.41] | 0.50 | NA | 0.01 [-0.02,0.04] | 0.56 | NA |
| MR-Egger | -0.78 [-2.08,0.51] | 0.27 | 6.40 (0.49) | 0.03 [-0.05,0.11] | 0.51 | 4.45 (0.81) |
| (MR-Egger intercept) | 0.09[-0.26,0.08] | 0.35 |  | -0.004 [-0.02,0.01] | 0.52 |  |
